# Metagenomic Analysis of the Enteric RNA Virome of Infants from the Oukasie Clinic, North West Province, South Africa, Reveals Diverse Eukaryotic Viruses

**DOI:** 10.3390/v12111260

**Published:** 2020-11-05

**Authors:** Milton T. Mogotsi, Peter N. Mwangi, Phillip A. Bester, M. Jeffrey Mphahlele, Mapaseka L. Seheri, Hester G. O’Neill, Martin M. Nyaga

**Affiliations:** 1 Next Generation Sequencing Unit, University of the Free State, Bloemfontein 9300, South Africa; tmogotsi16@gmail.com (M.T.M.); nthigapete@gmail.com (P.N.M.); 2 Department of Microbial, Biochemical and Food Biotechnology, University of the Free State, Bloemfontein 9300, South Africa; oneillhg@ufs.ac.za; 3 Division of Virology, School of Pathology, Faculty of Health Sciences, University of the Free State, Bloemfontein 9300, South Africa; besterpa@ufs.ac.za; 4 Diarrhoeal Pathogens Research Unit, Department of Virology, Faculty of Health Sciences, Sefako Makgatho Health Sciences University, Medunsa, Pretoria 0204, South Africa; Jeffrey.Mphahlele@mrc.ac.za (M.J.M.); mapaseka.seheri@smu.ac.za (M.L.S.); 5 South African Medical Research Council, 1 Soutpansberg Road, Pretoria 0001, South Africa

**Keywords:** RNA virome, rotavirus, viral metagenomics, enteric viruses

## Abstract

Establishing a diverse gut microbiota after birth is essential for preventing illnesses later in life. However, little knowledge exists about the total viral population (virome) present in the gut of infants during the early developmental stage, with RNA viruses being generally overlooked. Therefore, this small pilot longitudinal study investigated the diversity and changes in the enteric RNA virome in healthy infants from South Africa. Faecal samples (*n* = 12) were collected from four infants at three time points (on average at 8, 13, and 25 weeks), and then sequenced on an Illumina MiSeq platform. The genomic analysis revealed a diverse population of human enteric viruses from the infants’ stools, and changes in the enteric virome composition were observed over time. The *Reoviridae* family, more specifically the Rotavirus genus, was the most common and could be linked to viral shedding due to the administration of live-attenuated oral vaccines in South Africa, followed by the *Picornaviridae* family including parechoviruses, echoviruses, coxsackieviruses, enteroviruses, and polioviruses. Polioviruses were also linked to vaccine-related shedding. *Astroviridae* (astroviruses) and *Caliciviridae* (noroviruses) were present at low abundance. It is evident that an infant’s gut is colonized by distinct viral populations irrespective of their health state. Further characterization of the human virome (with a larger participant pool) is imperative to provide more conclusive insights into the viral community structure and diversity that has been shown in the current study, despite the smaller sample size.

## 1. Introduction

Enteric microbial colonization occurs at birth with exposure to microorganisms from the immediate environment. Establishing a diverse enteric microbiota in the early stages is imperative to prevent diseases later in life [1]. It has been noted that bacterial species increase in diversity during the early years of life due to both genetic and environmental factors including diet, pharmaceuticals, as well as lifestyle [2,3,4]. However, the viral populations within infants’ guts (enteric virome) remain less studied during this developmental stage [5]. Recent advances in next generation sequencing (NGS), have improved our understanding of the human gut virome [6,7]. Nevertheless, much remains unknown about the virome composition of healthy individuals [8].

The human virome consists of eukaryotic viruses, prokaryotic viruses, and endogenous viral elements integrated in the human genome [9]. While enteric virome has significant effects on human health, both in healthy and immune-compromised subjects, causing illnesses such as acute gastroenteritis (AGE) [10,11,12,13], there is still a considerable knowledge gap about the defined composition of the enteric viral communities [14]. Viral-induced AGE is responsible for childhood illnesses and deaths across the world [15]. Despite improvements in hygiene and prevention strategies, which significantly reduced the mortality rate due to diarrhea from 15% to 9% between 2008 and 2015 among children below the age of five years, infectious diarrhea is still a serious public health issue all over the world [15,16]. Interestingly, it is known that viruses are prevalent in the gastrointestinal tract, even in asymptomatic cases occurring as either latent pathogens or normal colonizing flora [14]. Findings from previous studies have suggested that most viral enteric infections were usually observed early in life rather than in adulthood, and such infections could be triggered by changes in the virome [17,18].

The application of metagenomics sequencing has become very useful in virome studies. However, these approaches have tended to overlook viral RNA genomes present in the virome, despite the fact that RNA viruses are implicated in most cases of AGE [17,19]. As a result, there is a great need to study RNA viruses, in order to establish and understand the dynamics of these RNA viral populations. The current pilot study aimed at assessing the viruses present in the gastrointestinal tract of infants at different stages of their lives. A metagenomics approach was employed to explore and characterize the enteric RNA virome of infants visiting the Oukasie clinic, in North West Province, South Africa. The faecal samples were collected from healthy children less than a year old. A diverse population of viruses, mostly associated with gastroenteritis, was detected in the faecal samples of all the infants, fluctuating with time. Despite the smaller sample size, this study has provided an overview on the diversity of viral agents colonizing an infant’s gut.

## 2. Materials and Methods

### 2.1. Sampling

This research study was conducted with the approval of the University of the Free State’s Health Sciences Research Ethics Committee (HSREC 130/2016 B (UFS-HSD2017/0374)). In this cohort study, twelve faecal samples were collected from four healthy South African infants (participants A, B, C, and D) attending check-ups at the Oukasie clinic, North West Province, South Africa, between March 2015 and November 2016. The study participants had each received two doses of Rotarix^®^ vaccine at 6 and 14 weeks. Samples were collected at three different time points, namely at approximately 8 weeks (baseline), 13 weeks (second cohort), and 24 weeks of age (third cohort). The samples were collected as part of the on-going intestinal microbiota profile surveillance study at the WHO Regional Reference Laboratory for Rotavirus, Diarrhoeal Pathogens Research Unit, Sefako Makgatho Health Sciences University (SMU), Pretoria, South Africa with ethical clearance number SMUREC/P/89/2015. The samples were, then, transferred to the University of the Free State’s Next Generation Sequencing (UFS-NGS) Unit, Bloemfontein.

### 2.2. Enrichment of RNA Virome

The NetoVIR protocol was used to process the enrichment of the viral particles for all twelve faecal samples [20], albeit with some in-house modifications. Briefly, 10% faecal suspensions were prepared in phosphate buffered saline (PBS), followed by homogenization and centrifugation. The supernatant was filtered through a 0.45 µm filter to remove eukaryotic and bacterial cell-sized particles but to retain larger RNA viruses. The resulting filtrate was subjected to nuclease treatment using Benzonase Nuclease (Sigma-Aldrich, St Louis, MO, USA) and Micrococcal Nuclease (Thermo Scientific, Waltham, MA, USA) in a buffer consisting of 1 M Tris, 100 mM CaCl_2_, and 30 mM MgCl_2_, pH 8, for 2 h, at 37 °C, to digest unprotected nucleic acids. The nuclease enzymes were inactivated with EDTA. Extraction of viral RNA was performed on viral particle enriched samples using a RNeasy PowerMicrobiome RNA isolation kit (Qiagen, Hilden, Germany). However, the bead-based homogenization step was bypassed since the enriched sample was already in liquid form. The eluted, purified, and quantified RNA was subjected to host ribosomal RNA (rRNA) removal using a NEBNext ribosomal RNA (rRNA) depletion (Human/Rat/Mouse) kit (New England Biolabs, Ipswich, MA, USA) following the manufacturer’s protocol.

### 2.3. Next Generation Sequencing

The purified viral RNA was subjected to reverse transcription and whole transcriptome amplification using a QIASeq FX Single Cell RNA Library Preparation Kit (Qiagen, Hilden, Germany). The amplified cDNA was assessed for quality using A260/A280 absorbance ratio on µlite Biodrop spectrophotometer (Biodrop, Cambridge, UK). The DNA library construction for next generation sequencing was done using a modified version of Nextera XT library preparation kit (Illumina, San Diego, CA, USA). The prepared libraries were sequenced on a MiSeq platform (Illumina), and 251 bp paired-end reads were generated using a V2 500 cycles kit.

### 2.4. Data Analysis

Using an in-house analysis pipeline, quality control of the generated reads was performed with FASTQc [21] and Prinseq-lite v0.20.4 [22]. Quality-filtered reads were de novo assembled using metaSPAdes [23]. Contigs were analyzed by BLASTX searched against the NCBI database using DIAMOND v0.9.22 [24], by aligning protein sequences against the NCBI protein database.

## 3. Results

### 3.1. Abundance of Viral Contigs

Following the processing of raw metagenomic data and analysis of the de novo assembled sequences, a total of 92,185 contigs were obtained from the 12 faecal samples. The BLASTX analysis of these contigs against the NCBI protein database revealed that 11.6% of the contigs mapped to viruses, translating to a total of 10,648 viral contigs. The abundance of viral contigs for the participants were as follows: participant A (585), participant B (9706), participant C (214), and participant D (143) (Table 1).

### 3.2. Taxonomic Classification of Viral Contigs

All the viruses detected in this study belonged to fourteen different virus families. The majority of the viral contigs belonged to the *Picornaviridae* family, constituting 93.7% of the total viral contigs. Nine different species within the *Picornaviridae* family were identified from various faecal samples in three of the four participants (A, B, and C) (Table 2). A large proportion of contigs (54.5%) were assigned to coxsackievirus A and B of the *Picornaviridae* family, detected across eight faecal samples. This was followed by vaccine-derived polioviruses (32.6%), comprising poliovirus 1, 2, and 3, detected across five samples, and the probable source was a trivalent oral polio vaccine (tOPV) given at birth. Other members of the *Picornaviridae* family that were detected included enterovirus A to D, echoviruses, parechoviruses, and rhinoviruses species (Table 2).

The family of *Reoviridae* was the second most detected, with 593 (5.56%) contigs detected from 12 samples. All these contigs belonged to group A rotaviruses, including G1P[8] genotypes as revealed by the BLAST search of randomly selected contigs (likely derived from the monovalent Rotarix^®^ rotavirus vaccine (RV) with genotype G1P[8] given from 6 weeks). Other virus families were only present in low abundance in the faecal samples and included, in decreasing order of contigs, *Retroviridae*, *Flaviviridae*, *Astroviridae,* and *Caliciviridae* (Table 2). In terms of classification based on genome structure, 28 viral species with different genomic structure were detected. These viruses included positive-sense single-stranded RNA (+ssRNA) viruses, negative-sense single-stranded RNA (−ssRNA) viruses, double-stranded RNA (dsRNA) viruses, DNA viruses, and other RNA viruses which could not be assigned into families (Table 3).

### 3.3. Frequency of Virus Detection and Abundance by Host Specificity

**Figure 1:**
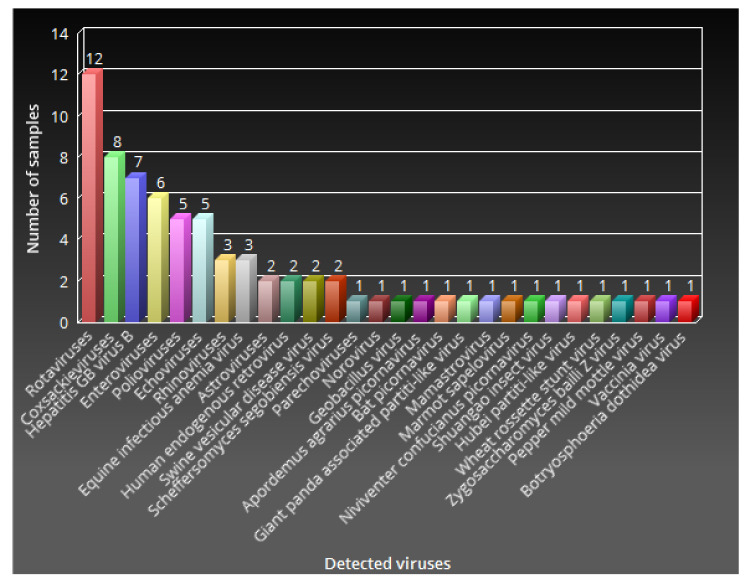
Detection rate of each virus among the twelve faecal samples. Only four different virus genera/species were present in at least half of the samples, with rotavirus being the only genus detected in all twelve samples. The majority of the viruses were present in three or fewer samples.

The evaluation of the frequency of viral detection (Figure 1) in the 12 faecal samples revealed a 100% detection rate for rotaviruses, followed by a 66.7% detection rate for coxsackieviruses, while enteroviruses and hepatitis GB virus B were detected in 58.3% and 50% of the samples, respectively. Polioviruses and echoviruses were detected in 41.17% of the samples. The rest of the viruses, including important human enteric pathogens such as noroviruses and astroviruses, were only detected in three samples or less (≥25%) (Figure 1).

**Figure 2:**
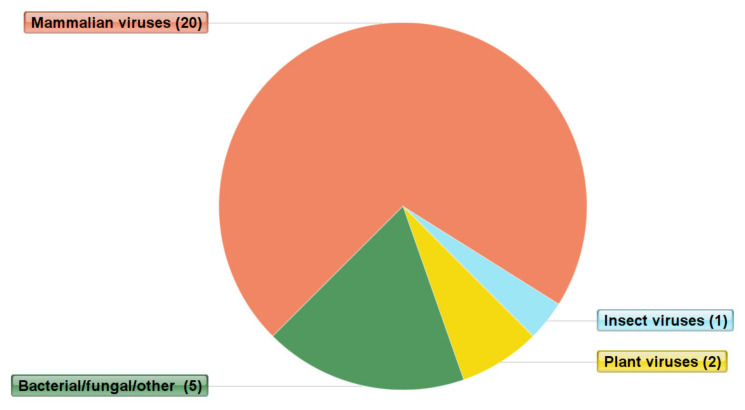
Different types of viruses from 12 faecal samples categorized based on host organism. These viruses included mammalian, which were the most predominant, followed by bacterial, fungal, others, and a small fraction of plant viruses and insect viruses.

Our analysis revealed that mammalian viruses were the most abundant, since 20 out of the 28 different viral genera/species detected, infected mammalian hosts including humans. The non-human mammalian viruses detected included the equine infectious anemia virus, swine vesicular disease virus, apodemus agrarius picornavirus, and niniventer confucianus picornavirus. Bacterial and fungal viruses, including a number of unclassified viruses, were the second most abundant, followed by two plant viruses, pepper-mild-mottle virus and wheat rosette stunt virus, detected in only one sample. Lastly, the shuangao insect virus was the only insect virus detected in one of the participants (Table 2 and Figure 2).

### 3.4. Distribution and Abundance of Mammalian Enteric RNA Viruses

**Figure 3:**
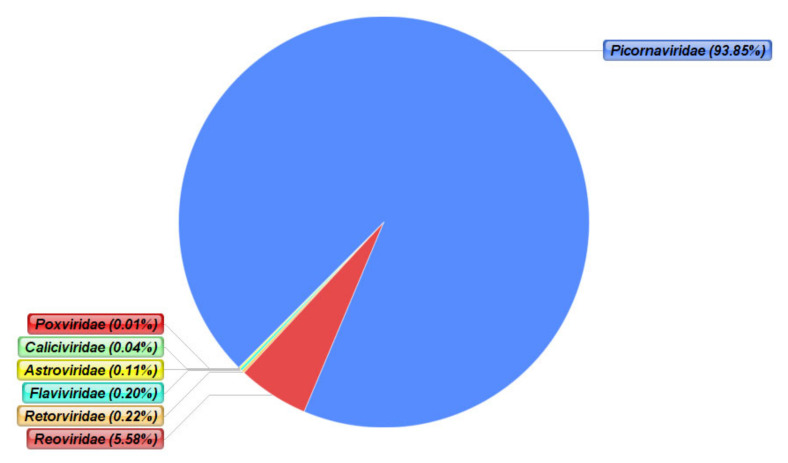
Percentages of contigs of the RNA viruses from all twelve faecal samples classified into their respective viral families. Over 90% of the viral contigs were classified under the *Picornaviridae* family. Other families present were *Reoviridae*, *Retroviridae*, *Flaviridae*, *Astroviridae*, *Caliciviridae*, and *Poxviridae*.

A total of seven different families of mammalian RNA viruses were detected in the faecal samples of the four study participants (Figure 3). Although *Picornaviridae* was detected in high numbers in terms of the overall viral contigs, *Reoviridae* was the most common family, as it was present in all 12 faecal samples.

**Figure 4:**
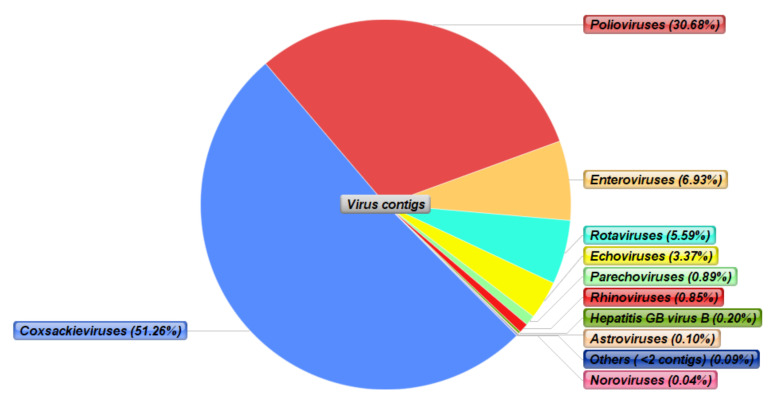
Sequence (contigs) distribution of the detected mammalian RNA viruses at the genus/species level from twelve faecal samples. Coxsackieviruses and polioviruses were the two most abundant species, contributing to 51.26% and 30.68% of the virus contigs, respectively.

Further analysis that examined the distribution of viral contigs at the genus or species level, and focused specifically on mammalian RNA viruses, showed that nearly 50% of the viral RNA contigs were derived from coxsackieviruses (Figure 4), followed by polioviruses, with nearly 30% of the viral contigs mapping to this species of positive-sense single-stranded RNA virus. Furthermore, enteroviruses and rotaviruses consisted of 6.93% and 5.59% of the viral contigs, respectively. Echoviruses were the fifth most abundant viruses with 3.37%, whilst the remaining five families consisted of less than 1% of the viral contigs. Interestingly, one of the most important human enteric pathogens, i.e., norovirus, was detected, although there were only four contigs that mapped to this species.

### 3.5. Gut Virome Composition and Changes over Time for Each Study Participant

We analyzed the change in viral populations over time for all four study subjects, focusing specifically on the RNA viruses detected from each of the four infant’s faecal samples. In participant A, there was a fluctuation in the number of viral contigs, particularly for rotavirus over the three collection time points. During the first collection (6 weeks), there were 58 rotavirus contigs, which then dropped to 27 contigs during the second collection time (10 weeks), and another increase to 59 viral contigs was observed by the last collection (36 weeks) (Table 2). Interestingly, during the last collection (36 weeks), a dramatic increase was observed in the number of viral contigs for coxsackievirus (84 contigs), from only three contigs in the previous collections combined. Another interesting observation was the emergence of two other viruses, echovirus (309) and enterovirus (34), during the last collection time point.

In the case of participant B, an increase in the number of viral contigs was observed between the first and second collection, followed by a drastic decline during the final collection. At a baseline of 10 weeks, coxsackievirus was the most frequently detected in this infant (1386 contigs), followed by polioviruses (914 contigs), then enteroviruses and rotaviruses with 146 and 51 viral contigs, respectively (Table 2). Echoviruses and astroviruses were detected in low quantities, five and two contigs, respectively. During the second collection (14 weeks), a rise in the number of contigs was observed for most of the viruses, with several other viruses emerging (Table 2). The highest number of viral contigs detected during the second collection (14 weeks) were observed in polioviruses, with a sharp increase from 914 to 2333 contigs, while another increase was also observed in coxsackieviruses from 1386 to 1953 contigs. Enteroviruses and rotaviruses followed with 549 and 90 contigs, respectively. Remarkably, there was a significant decline in the number of viral contigs during the last collection time point (24 weeks) for most of the viruses that were detected in this infant, and some viruses such as enteroviruses and rhinoviruses were completely cleared by the last collection. (Table 2). There were only two contigs for coxsackievirus (decreasing from 1953 contigs), while only four contigs for polioviruses could be detected during the final collection. Although there was a decrease in rotavirus contigs, a significant number of contigs (22) were still present in the sample during the third collection. Surprisingly, there was a sudden emergence of parechoviruses during the third time point with a total of 94 contigs being detected (Table 2).

In the case of study participant C, the majority of the viruses presented with a minimal number of contigs, with only rotavirus being present in abundance. At baseline (6 weeks), 52 contigs were attributable to rotaviruses and 20 contigs assigned to echoviruses, while the other viruses had contigs ranging from one to five (Table 2). During the second collection time point (14 weeks) and third (20 weeks) collection time point, only rotavirus contigs were present in high frequencies in participant C (Table 2).

Participant D presented the least number of viruses as compared with the other three study participants. Similarly, rotavirus was the most abundant, increasing progressively over time from 32 contigs at baseline (six weeks); to 67 contigs detected during the last collection time point (Table 2).

## 4. Discussion

This study aimed to characterize the taxonomic composition of the enteric RNA virome of four South African infants. According to the *de novo* assembly and BLASTX annotation using DIAMOND, 11.6% of the assembled contigs mapped to viruses, the majority of which were eukaryotic RNA viruses. *Picornaviridae* constituted the highest number of viral contigs with coxsackieviruses and polioviruses being the most abundant within this viral family, even though these viruses were not the most commonly detected across all samples. Only one of the four infants (participant B) presented significant quantities of poliovirus contigs in the faecal samples (Table 2). The drastic decline in poliovirus contigs, at 24 weeks, correlated with previous reports that showed, after exposure to the oral poliovirus vaccine, immuno-competent individuals shed poliovirus vaccine strains for a short period of time, often for less than two months [25,26]. To the contrary, several immuno-deficient OPV recipients have been reported to excrete polioviruses for several years [25,27,28].

Human enteroviruses, within the *Picornaviridae* family, commonly transmitted via the faecal-oral route and occasionally by respiratory droplets [29,30] were also among the three viruses with a high number of contigs. Enteroviruses cause a range of illnesses including respiratory infection, hand-foot-and-mouth disease, and aseptic meningitis [31,32]. A recent study reported meningitis outbreaks linked to human enteroviruses in South Africa [33], which followed an earlier report of enterovirus-associated meningitis in young children in other parts of South Africa [34]. However, the clinical role of enteroviruses in these infants remains unknown, as no clinical data were available for any of the four study participants.

Group A rotaviruses, the leading cause of acute gastroenteritis in young children, were detected in all 12 samples (100%), making it the most frequently detected genus in this study. Group A rotaviruses have been responsible for 128,500 deaths occurring every year among children under five years old, especially in the developing countries of sub-Saharan Africa and Asia [35]. Although no prevalence studies have been conducted at the genotype level for rotaviruses, secondary BLAST analysis of randomly selected rotavirus contigs has revealed the presence of G1P[8] strains among others, suggesting the presence of the live-attenuated vaccine, Rotarix^®^ (GlaxoSmithKline, Rixensart, Belgium), which contained the same genotype. Rotarix^®^ is a two-dose scheduled live-attenuated human rotavirus oral vaccine administered in South Africa to infants at six and 14 weeks [36]. Vaccine strain shedding is often described [37].

Other viruses detected at low frequency from the infants’ stools were astrovirus and norovirus. These are important and well-characterized viral agents that have been implicated in cases of childhood diarrhea. Although this gut viral metagenomic study involved healthy infants, norovirus has been known as the most common agent of acute gastroenteritis in humans of all ages worldwide [38]. However, subclinical infections, resulting in undiagnosed infections, have also been reported [39].

A few contigs mapping to bacteriophages and plant viruses have also been identified. The detection of bacteriophages and plant viruses in the human gut virome has been described before [40,41]. Only one sample, each from participants B and C, contained one plant virus, namely pepper mild little virus and wheat rosette stunt virus, respectively (Table 2). Coincidentally, the two viruses were detected at the same age (14 weeks old) in the respective infants. Pepper mild mottle virus, which is a well-characterized plant pathogen that infects all species in the genus *Capsicum*, has previously been reported to be present in abundance in the faeces of healthy humans [19]. These plant viruses have never been associated with any human disease. The presence of plant viruses in stool samples of humans is often a reflection of acquisition from diet. However, diet is an unlikely source in the current study. The parents or guardian, fomites, or immediate environment are the more likely sources.

An assessment of the temporal dynamics of viral populations in participant A revealed the detection of nine different viruses, however, among these, only coxsackieviruses and rotaviruses were present throughout the collection period. The viral sequences were mostly detected during the last collection time point, at 36 weeks (equivalent to nine months). Interestingly, and in correlation with our findings, a related study that longitudinally investigated the enteric virome composition and dynamics of healthy infants (from birth to 24 months old), from USA, reported that eukaryotic viral population richness of an infant’s gut was low early in life and increased thereafter. According to this observation, the assumption was that the eukaryotic virome was established through environmental exposure [42]. It is important to mention that this observation was made only in one infant (participant A), but not in the other three infants. Moreover, in the USA study, enteroviruses and parechoviruses were among the most commonly detected viruses and, since similar viral sequences were also detected in our study, these findings could suggest that these enteric viruses form part of the viral flora in the gut of healthy infants.

Although human rhinoviruses, single-stranded RNA viruses within the *Picornaviridae* family, are considered respiratory pathogens, since it has been reported that their replication in the intestinal mucosa is hampered by their sensitivity to low pH, our study and other studies have reported their detection in faecal samples of young children [43,44,45].

Apart from the above-mentioned human viruses discussed so far, a few sequences that mapped to non-human mammalian viruses were also detected in the stool samples of participant C. These included equine infectious anemia virus, a horse-infecting viral pathogen from the family *Retroviridae* commonly transmitted by blood-sucking insects such as horse fly and deer fly [46]. The other virus detected was swine vesicular disease virus, a pig enterovirus from the family *Picornaviridae*. The latter was previously reported to be a porcine variant of human coxsackievirus B5 [47], a member of the *Picornaviridae* which was detected in majority of the samples in this study.

In the current study, participant B presented the most diverse population of viruses, most of which were eukaryotic single-stranded RNA viruses. This infant exhibited a distinctive pattern over the three collection time points, whereby there was a significant increase in the viral contigs between 10 and 14 weeks for certain viruses including coxsackieviruses, rotaviruses, echoviruses, enteroviruses, astroviruses, polioviruses, rhinoviruses, and noroviruses. A decline in the viral contigs was observed at 24 weeks, with some of the viruses undetected. Among the viruses detected in participant B, there were several non-human eukaryotic viruses including unclassified members of the order *Picornavirales*, namely apodemus agrarius picornavirus and niniventer confucianus picornavirus. The former is known to infect striped field mice, whilst the latter infects a species of rodents in the *Muridae* family. Other non-human eukaryotic virus sequences recovered in participant B included bat picornavirus, marmot sapelovirus, genus Mamastrovirus, giant panda-associated partiti-like virus, swine vesicular disease virus, and shuangao insect virus. The detection of these viruses, especially from field animals could be due to indirect environmental exposure, considering that Oukasie is a small, poor Township in the rural North West Province of South Africa with unavailability of proper sanitation.

Additionally, parechovirus was the only virus found exclusively in participant B, which has been previously detected in asymptomatic subjects. More specifically, a case-control study was done in Ghana to determine the prevalence of human parechoviruses, as well as its association with diarrhea in children [48]. The study reported a high detection rate and diversity of human parechovirus in asymptomatic controls. However, in our study, the detection rate of parechoviruses was low (1/12 samples), although the sample size was small.

Unlike participant B, relatively low diverse viral populations were observed in participant C. The same enteric viruses that were described in the previous two participants were also present in participant C, most of which belonged to the *Picornaviridae* and *Reoviridae* families.

Human endogenous retrovirus has commonly been found integrated into the host genome, and sequences of this virus were discovered in the first and the last collected samples of participant D. In particular, participant D was one of the two infants that had a DNA virus and the only infant in which a bacteriophage sequence was recovered (Geobacillus virus).

This study has provided a description of the enteric virome of infants from Oukasie Township. To our knowledge, it represents the first cohort metagenomics characterization of the RNA enteric virome of infants from South Africa. We have demonstrated that even in asymptomatic cases, the human gastrointestinal tract is still colonized by diverse populations of eukaryotic viruses. Furthermore, taking into account the enormous size and diversity of viral populations that have been discovered thus far, very often a significant proportion of the sequences obtained from metagenomic studies of the human virome cannot be annotated and classified into taxonomic groups. There are only a few thousand viral reference sequences that exist in genome databases, implying that any potential new virus obtained from the human or environmental sample will usually lack resemblance to genomes in the databases.

## 5. Conclusions

The human enteric virome remains largely unexplored. Several drawbacks, such as the financial load of metagenome studies, a limited number of study participants, unavailability of clinical information, and inadequate analysis tools, make it difficult to draw conclusions from metagenome studies. The sequence databases currently available are known to contain sequence information of known viruses, especially those that are deemed clinically and economically relevant. As a result, it becomes challenging to assign viruses recovered from metagenomic studies into viral families. Although viral metagenomic methods can provide insights into the composition and structure of viral communities colonizing the gastrointestinal tract of humans, factors such as diet, nutritional status, immunization status with live attenuated vaccines, geographical location, health including immune status, and socioeconomic group should be taken into consideration, as these can influence the composition of the human gut virome. In summary, there is a great need for thorough screening of infants and young children in order to establish the prevalence and diversity of viruses colonizing their gut system. These efforts could allow for efficient measures and prevention of viral-induced illnesses including diarrhea in children.

## Figures and Tables

**Table 1:** Summary of the distribution of assembled contigs obtained from the twelve faecal samples of the four study participants.

Sample	Collection Time	Total Assembled Contigs	Viral Contigs	Percentage Viral Contigs
A1	6 weeks	5549	68	1.2
A2	10 weeks	6255	28	0.4
A3	36 weeks	9805	489	5.0
Total		21,609	585	2.7%
B1	10 weeks	12,445	2504	20.1
B2	14 weeks	15,184	7064	46.5
B3	24 weeks	5636	138	2.4
Total		33,265	9706	29.2%
C1	6 weeks	4368	87	2.0
C2	14 weeks	8637	60	0.7
C3	20 weeks	3638	67	1.8
Total		16,643	214	1.3%
D1	6 weeks	7405	36	0.5
D2	14 weeks	7357	37	0.5
D3	20 weeks	5906	70	1.2
Total		20,668	143	0.7%
Overall		92,185	10,648	11.6%

**Table 2:** Taxonomic distribution of detected viruses from assembled contigs per collected faecal sample.

Virus Family					*Picornaviridae*									*Reoviridae*	*Astroviridae*		*Retroviridae*		*Caliciviridae*	*Flaviviridae*	*Poxviridae*	*Virgaviridae*	*Rhabdoviridae*	*Siphoviridae*	*Amalgaviridae*	*PartitiviridaE*	*Totiviridae*	*Peribunyaviridae*	Unclassified *picornavirales*		Unclassified	
SAMPLE NUMBER	Sample ID	Sample Collection Time (Weeks)	Total Number of Contigs	Contigs Assigned to Viruses	Coxsackievirus A, B	Enterovirus A, B, C, D	Echovirus E	Poliovirus 1, 2, 3	Parechovirus 1, 3, 8, 17, 19	Rhinovirus A, B, C	Swine vesicular disease virus	Bat picornavirus	Marmot sapelovirus	Rotavirus A	Astrovirus	Mamastrovirus	Human endogenous retrovirus	Equine infectious anaemia virus	Norovirus GI, GII.4	Hepatitis GB virus B	Vaccinia virus	Pepper mild mottle virus	Wheat rosette stunt virus	Geobacillus virus	Zygosaccharomyces bailli Z virus	Botryosphoeria dothidea virus	Scheffersomyces segobiensis virus	Shuangao insect virus	Apodemus agrarius picornavirus	Niniventer confucianus picornavirus	Hubei partiti-like virus	Giant panda associated partiti-like virus
Genomic Structure					(+)ssRNA									dsRNA	(+)ssRNA		RNA	(+)ssRNA	(+)ssRNA	(+)ssRNA	dsDNA	(+)ssRNA	(−)ssRNA	dsDNA	dsRNA	dsRNA	dsRNA	(−)ssRNA	(+)ssRNA	(+)ssRNA	RNA	RNA
**A1**	4450	6	5549	**68**	**2**	0	0	0	0	1	0	0	0	**58**	0	0	0	**1**	0	**2**	0	0	0	0	0	0	**5**	0	0	0	0	0
**A2**	6618	10	6255	**28**	**1**	0	0	0	0	0	0	0	0	**27**	0	0	0	0	0	0	0	0	0	0	0	0	0	0	0	0	0	0
**A3**	8908	36	9805	**489**	**84**	**34**	**309**	0	0	0	**2**	0	0	**59**	0	0	0	0	0	0	0	0	0	0	0	0	0	0	0	0	0	0
**B1**	8903	10	12,445	**2504**	**1386**	**146**	**5**	**914**	0	0	0	0	0	**51**	2	0	0	0	0	0	0	0	0	0	0	0	0	0	0	0	0	0
**B2**	8941	14	15,184	**7064**	**3953**	**549**	**22**	**2333**	0	**88**	**3**	**1**	**1**	**90**	**9**	**1**	0	**1**	0	**5**	0	0	0	0	0	0	**2**	**1**	**1**	**1**	0	**1**
**B3**	10,287	24	5636	**138**	**2**	**1**	0	**4**	**94**	0	0	0	0	**20**	0	0	0	0	**4**	**6**	**1**	**1**	0	0	0	0	**3**	0	0	0	**1**	0
**C1**	8824	6	4368	**87**	**5**	**4**	**20**	**1**	0	0	0	0	0	**52**	0	0	0	0	0	**3**	0	0	0	0	0	**1**	0	0	0	0	0	0
**C2**	10,127	14	8637	**60**	**2**	0	0	**1**	0	0	0	0	0	**57**	0	0	0	0	0	0	0	0	0	0	0	0	0	0	0	0	0	0
**C3**	10,233	20	3638	**67**	0	**1**	**1**	0	0	**1**	0	0	0	**43**	0	0	0	**17**	0	**2**	0	0	**1**	0	**1**	0	0	0	0	0	0	0
**D1**	8355	6	7405	**36**	0	0	0	0	0	0	0	0	0	**32**	0	0	**1**	0	0	**2**	0	0	0	**1**	0	0	0	0	0	0	0	0
**D2**	8910	14	7357	**37**	0	0	0	0	0	0	0	0	0	**37**	0	0	0	0	0	0	0	0	0	0	0	0	0	0	0	0	0	0
**D3**	10,098	20	5906	**70**	0	0	0	0	0	0	0	0	0	**67**	0	0	**2**	0	0	**1**	0	0	0	0	0	0	0	0	0	0	0	0
**TOTAL VIRUS CONTIGS**				**10,648**	**5435**	**735**	**357**	**3253**	**94**	**90**	**5**	**1**	**1**	**593**	**11**	**1**	**3**	**19**	**4**	**21**	**1**	**1**	**1**	**1**	**1**	**1**	**10**	**1**	**1**	**1**	**1**	**1**

**Table 3:** Different types of viruses detected from twelve faecal samples categorized based on the genome type.

Sample Number	Collection Time	(+)ssRNA Viruses	(−)ssRNA Viruses	dsRNA Viruses	DNA Viruses	Unclassified RNA Viruses	Total (Different Viral Genomes)
**A1**	6 weeks	3	-	2	-	-	5
**A2**	10 weeks	1	-	1	-	-	2
**A3**	36 weeks	5	-	1	-	-	6
**B1**	10 weeks	5	-	1	-	-	6
**B2**	14 weeks	13	1	2	-	1	17
**B3**	24 weeks	8	-	2	1	1	12
**C1**	6 weeks	5	-	2	-	-	7
**C2**	14 weeks	2	-	1	-	-	3
**C3**	20 weeks	5	1	2	-	-	8
**D1**	6 weeks	1	-	1	1	1	4
**D2**	14 weeks	-	-	1	-	-	1
**D3**	20 weeks	1	-	1	-	1	3
